# The 10 “Cardinal Sins” in the Clinical Diagnosis and Treatment of Endometriosis: A Bayesian Approach

**DOI:** 10.3390/jcm12134547

**Published:** 2023-07-07

**Authors:** Philippe R. Koninckx, Anastasia Ussia, Stephan Gordts, Jörg Keckstein, Ertan Saridogan, Mario Malzoni, Assia Stepanian, Antonio Setubal, Leila Adamyan, Arnaud Wattiez

**Affiliations:** 1 Department of OBGYN, Faculty of Medicine, Katholieke University Leuven, 3000 Leuven, Belgium; 2 Department of OBGYN, Faculty of Medicine, University of Oxford, Oxford OX1 2JD, UK; 3 Department of OBGYN, Faculty of Medicine, University Cattolica, del Sacro Cuore, 00168 Rome, Italy; 4 Department of OBGYN, Faculty of Medicine, Moscow State University, 119991 Moscow, Russia; 5 Latifa Hospital, Dubai 9115, United Arab Emirates; arnaud.wattiez@wanadoo.fr; 6 Department of OBGYN, Gemelli Hospitals, Università Cattolica, 00168 Rome, Italy; anastasia.ussia@gmail.com; 7 Leuven Expert Center, 3000 Leuven, Belgium; stephan.gordts@lifeexpertcentre.be; 8 Endometriosis Centre, Dres. Keckstein, 9500 Villach, Austria; joerg@keckstein.at; 9 Faculty of Medicine, University Ulm, 89081 Ulm, Germany; 10 Elizabeth Garrett Anderson Institute for Women’s Health, University College London, London SW7 2BX, UK; ertan.saridogan@gmail.com; 11 Malzoni Centre Avelino, 83100 Avellino, Italy; malzonimario@gmail.com; 12 Academia of Women’s Health and Endoscopic Surgery, Atlanta, GA 30328, USA; astep@migsurgery.com; 13 Department of Ob/Gyn and MIGS, Hospital da Luz Lisbon, 1500-650 Lisboa, Portugal; asetubal@hospitaldaluz.pt; 14 Department of Operative Gynecology, Federal State Budget Institution V. I. Kulakov, Research Centre for Obstetrics, Gynecology, and Perinatology, Ministry of Health of the Russian Federation, 117198 Moscow, Russia; adamyanleila@gmail.com; 15 Department of Reproductive Medicine and Surgery, Moscow State University of Medicine and Dentistry, 127473 Moscow, Russia; 16 Department of Obstetrics and Gynaecology, University of Strasbourg, 67081 Strasbourg, France

**Keywords:** endometriosis diagnosis, endometriosis therapy, endometriosis surgery, Bayesian statistics, evidence-based medicine

## Abstract

Evidence-based data for endometriosis management are limited. Experiments are excluded without adequate animal models. Data are limited to symptomatic women and occasional observations. Hormonal medical therapy cannot be blinded if recognised by the patient. Randomised controlled trials are not realistic for surgery, since endometriosis is a variable disease with low numbers. Each diagnosis and treatment is an experiment with an outcome, and experience is the means by which Bayesian updating, according to the past, takes place. If the experiences of many are similar, this holds more value than an opinion. The combined experience of a group of endometriosis surgeons was used to discuss problems in managing endometriosis. Considering endometriosis as several genetically/epigenetically different diseases is important for medical therapy. Imaging cannot exclude endometriosis, and diagnostic accuracy is limited for superficial lesions, deep lesions, and cystic corpora lutea. Surgery should not be avoided for emotional reasons. Shifting infertility treatment to IVF without considering fertility surgery is questionable. The concept of complete excision should be reconsidered. Surgeons should introduce quality control, and teaching should move to explain why this occurs. The perception of information has a personal bias. These are the major problems involved in managing endometriosis, as identified by the combined experience of the authors, who are endometriosis surgeons.

## 1. Introduction

The diagnosis and treatment of endometriosis should be based on the best evidence available, as discussed in many reviews [1,2,3,4,5]. Evidence-based medicine (EBM) emphasises the importance of avoiding allocation, patient or observer bias, and (traditional) statistical validation. The translation into guidelines [6,7,8,9,10,11,12] requires interpreting the quality of the evidence in the literature, trials, and clinical case series. This clinical translation explains that guidelines such as those from ACOG, ASHRE, ASRM, and JOGC can differ [7,13,14], requiring tools like AGREE-II [15] to evaluate the guidelines’ quality, and why the implementation of EBM in clinical practice proved difficult [16,17]. 

Besides the problematic translation of evidence into clinical grades, EBM has not (yet) fully recognised the limitations of frequentist or traditional statistics [18,19], which can refute, but not confirm, a hypothesis. Using a significant result as an argument with which to confirm a hypothesis is a frequent mistake in medicine, known as the *p*-value fallacy [20]. Awareness of this problem is still limited, despite the statement of the American Statistical Association [21] in 2016 and the indirect Bayesian conclusions that the majority of published data in medicine must be wrong [22,23]. Another type of statistical analysis is needed to confirm a hypothesis or calculate the probability that a hypothesis is true [24]. However, the use of Bayesian statistics is still limited in medicine and EBM guidelines. Less recognised is that traditional statistical hypothesis testing assumes that the data come from a homogeneous population and that traditional statistics rarely detect smaller subgroups. This can be problematic for the diagnosis and treatment of endometriosis, since endometriosis is biochemically heterogeneous [25]. In addition, subtle, typical, cystic, and deep endometriosis might be genetically or epigenetically different diseases [26]. Therefore, it cannot be concluded that the results of medical therapy apply to all subgroups, such as those with progesterone resistance [27]. A third problem with EBM is that randomised controlled trials (RCTs) are poorly suited for analysing rare events or multimorbidity unless datasets are sufficiently large to have substantial numbers of these rare cases. Therefore, the conclusions of trials address only the largest group and not necessarily the entire population, since rare events occurring less than 10 or 30 times are ignored. 

Especially for endometriosis, an evidence-based diagnosis and treatment are problematic, since solid evidence is poorly available. The absence of an appropriate animal model prevents experimentation. Epidemiology is poorly known [28], since the diagnosis is restricted to symptomatic women without reliable non-invasive diagnoses. Medical therapy cannot be blinded when recognised by the patient. The RCTs of severe surgery are unrealistic given the high clinical and biochemical variability combined with a relatively small number of cases [18]. Clinical judgment varies with subspecialties, with a degree of commercialisation and industrialisation being involved [29]. 

Clinical medicine and research are fundamentally different. First, the RCT estimates the probability of an effect without considering its magnitude, resulting in a publication bias where tiny differences become “significant” because of large numbers [30]. Secondly, clinical medicine diagnoses and treats all types of endometriosis in women of all ages, including women with multimorbidity. This differs from randomised controlled trials (RCT), which limit variability by inclusion and exclusion criteria, and, thus, have an extrapolation problem. Third, the RCT is not suited to addressing several parameters simultaneously, and clinical medicine is generally multivariate. For example, clinicians may decide to perform surgery on cystic ovarian endometriosis by combining parameters such as the size of the lesions, pain, the age of the woman, eventual infertility, the risk of cancer, and the risk of missing other diagnoses. Finally, each diagnosis and treatment method can be considered an experiment with an outcome used by the clinician to improve the subsequent diagnosis and treatment. This process is the personal clinical experience, which consists of a progressive Bayesian management update. Unfortunately, EBM considers this personal experience a “personal opinion”, of low value in the pyramid of evidence, because of the many potential biases [31]. 

We only recently began to appreciate the value of the similar personal experiences of many surgery-oriented endometriosis specialists [16,32]. As a proof of concept, we recently demonstrated how this collective experience of over 10,000 instances of endometriosis management can be documented for a series of statements [33]. These results constitute a “Bayesian prior” and will permit the calculation of a probability of truth when new data become available. After that, these data are expected to help to update the guidelines. 

The collective-experience-based method and EBM are complementary. EBM is the basis on which experience is built, but the collective experience extrapolates EBM to the entire population, including rare events and their multimorbidities. It also comprises our mistakes, complications, pitfalls, observations, and near-misses. Collective experience also addresses issues which cannot be addressed in an RCT. An RCT can address the result of a treatment, but cannot address what should not be done or what should be avoided. The knowledge of what should not be done constitutes valuable collective experience-based wisdom. To prepare for a formal investigation of these collective experience-based mistakes in diagnosing and treating endometriosis, we decided to describe the perception of the most frequent mistakes and challenges in endometriosis management, called the “cardinal sins” by deep endometriosis surgeons. 

## 2. Materials and Methods

The cumulative experiences of over 50,000 treatments of women with endometriosis were estimated as the sum of the individual experiences of the authors (PK > 5000, AU> 3000, SG > 5000, JK > 5000, MM > 5000, AS > 1000, SA > 1000, LA > 5000, AW > 5000) and those acknowledged (BA 500, PT 4000, HF 4000, WK > 5000, PA > 5000, GC > 1000). The included doctors all serve at referral centres for women with endometriosis. They also treat patients with previous surgical or medical treatments, allowing them to learn from each patient’s past and current experiences. These data reflect the authors’ discussions during events, meetings, and live surgeries. The text was also sent to a group of younger surgeons at Latifa Hospital and to the Winner’s groups, asking them to be listed in acknowledgements indicating their agreement or disagreement. The list of mistakes to avoid was limited to 10 items in an attempt to cover the most important concepts. That all authors be surgery-oriented was not a bias, but a pre-requisite to reflect the collective experience of surgery-oriented gynaecologists. However, these observations might differ and should be complemented by clinicians with different sub-specialities. The sequence of descriptions does not indicate importance. We reviewed Pubmed and could not find a single article describing what should be avoided in the diagnosis and treatment of endometriosis. 

## 3. Results: The 10 “Cardinal Sins”

### 3.1. To Consider Endometriosis as One Homogeneous Disease

Endometriosis can no longer be considered as endometrium implanted outside the uterus [27], since the lesions are clonal and macroscopically and biochemically different, e.g., with variable aromatase activity and progesterone resistance. This can be explained by the genetic–epigenetic theory [26], which postulates that endometriosis lesions begin their development only after a cumulative number of genetic or epigenetic incidents have exceeded a certain threshold, changing the endometrium cell into an endometriotic cell, as supported by the different lipid profiles [34] and gene expression [35]. The predisposition to and heredity of endometriosis thus reflect the risk of exceeding this threshold, and the risk increases when the inherited or in utero-imprinted incidents are already numerous or important. This predisposition is believed to be reflected clinically in infertility, changes in the junctional zone [36], and severe dysmenorrhoea from the first menstruation onwards [37], even before endometriosis lesions have formed. Another consequence of this predisposition is the high risk of initiating endometriosis lesions soon after puberty [38], when the oxidative stress of retrograde menstruation or infection or the microbiome [39] increases the risk of additional incidents. 

To consider endometriosis as the consequence of a series of genetic or epigenetic incidents changes our perspective on prevention. Daughters of women with endometriosis might deserve specific attention, especially when maternal endometriosis is severe. Although unproven today, recommending fruits and vegetables as anti-oxidants seems logical and free of risk. Also, the vaginal microbiome deserves more attention, with strict therapy and follow-up for infections. A reduction in retrograde menstruation will decrease peritoneal oxidative stress, but medical treatment to abolish menstruation seems too invasive to be recommended without trial evidence. 

Recognising the biochemical heterogeneity of endometriosis lesions is important when treating endometriosis with medical therapy. This heterogeneity explains that medical therapy is highly effective in treating pain in some 70% [40] of women, but has no or little effect in 10% and 20%, respectively. This also explains the need for a strict follow-up, e.g., with imaging, during therapy, since some lesions can continue growing and new lesions may develop. For the same reason, a more invasive diagnosis by laparoscopy, preferably by expert endometriosis surgeons, seems logical in women with insufficient pain relief after 3 to 6 months. Heterogeneity also explains that some endometriosis lesions can grow despite low plasma oestrogen concentrations, as demonstrated by severe deep endometriosis lesions [41] after menopause. 

As clinicians, we, therefore, suggest considering the diagnosis of endometriosis and potential preventive measures early in the adolescent daughters of women with endometriosis. We recommend to reconsider medical treatment for endometriosis if its effectiveness in reducing pain is limited, to monitor the growth of endometriosis lesions during medical therapy, and to consider the presence of severe endometriosis and rare primary peritoneal malignancy, also after menopause, if clinical symptoms are suggestive of it.

### 3.2. Inaccurate Judgement of the Diagnostic Accuracy of Imaging and Clinical Exam 

The sensitivity and specificity of ultrasound and MRI imaging for diagnosing cystic ovarian and deep endometriosis are established test characteristics. However, clinicians need to determine the probability that a positive test result will indicate that a woman has endometriosis, as well as the risk of missing the diagnosis if the test is negative. These are the positive and negative predictive values, and clinicians should be aware of the many pitfalls when translating sensitivity and specificity into predictive values. 

First, a negative exam cannot rule out superficial, cystic, or deep endometriosis, since the lower detection limits have not yet been established [42]. Second, imaging accuracy cannot distinguish reliably between cystic ovarian endometriosis and a cystic corpus luteum. Therefore, surgery should be postponed when imaging and clinical signs such as an acute onset of pain or a mobile cystic ovary could suggest a cystic corpus luteum or other non-concerning cystic pathology. The duration of the persistence of a cystic corpus luteum or other non-concerning cystic pathologies during ovarian suppression is not known. Still, we have observed persistence for more than six months. Third, a test’s predictive value decreases sharply when the disease’s prevalence is below 10%. Therefore, the PPV of deep endometriosis, with a prevalence of only a few per cent, is hardly higher than 50% to 70% [42] unless performed in a referral centre with a prevalence of above 10%. Fourth, without blinding the surgeon to the imaging results, it cannot be concluded through trial evidence that imaging predicts the type or extent of the necessary surgery [18]. However, in endometriosis management, imaging and a classification system such as #Enzian are important and useful for predicting surgical difficulty, counselling the patient, and guiding surgery [43,44]. We agree that a contrast enema demonstrating more than 50% sigmoid stenosis over more than 2 cm indicates the need for a sigmoidal resection anastomosis. Also, a deep endometriosis lesion of more than 3 × 3 × 3 cm or a lesion with a volume of more than 20 mL will require a bowel resection or a debulking, followed by a wedge resection with a circular stapler, in the large majority of women. The clinical combination of symptoms, history, examination, imaging, and laboratory data will enhance the accuracy of diagnosis, suggest conducting or postponing an intervention, and estimate the extent of the necessary surgery. 

### 3.3. Medical Therapy to Avoid Surgery in Scenarios Requiring Surgical Intervention

Medical therapy, generally, is the first treatment in women with endometriosis-associated pain. Surgery should be considered in women with severe pain, insufficient pain relief, or endometriosis lesions that grow during medical treatment. However, surgeons need to recognise the extent of the disease and estimate surgery based on clinical data, imaging, and the patient’s goals. Surgeons then need to decide whether they have the skills to perform the surgery effectively and safely and, if needed, to refer the patient or obtain help to provide complete surgical services. Surgery can be unexpectedly challenging, and experience is necessary in order to recognise endometriosis and to perform excision without complications. Without discussing the skills required for each type of surgery, the non-expert surgeon faces the choice of referring the patient or risking being confronted with surgery too difficult for their skills. 

When surgery is expected to be difficult and complication-prone, clinicians might prefer to postpone surgery and continue medical treatment despite incomplete pain relief. The consequences are unnecessary suffering of the patient and the development of possibly more extensive and more severe endometriosis lesions. In our collective clinical experience, we have frequently observed severe and technically challenging deep endometriosis surgery in women who had taken medical treatment for more than 5 or 10 years, even with reasonable pain control. 

During adolescence, the clinician’s concern is that endometriosis might grow, even during medical therapy, and more severe lesions might develop. The dilemma of surgeons regarding a non-severely symptomatic adolescent is allowing for the potential growth of the lesions despite medical treatment versus early excisional laparoscopy, which carries the potential risk of recurrences needing repeat surgery, along with the risk of adhesion formation. Unfortunately, there are only anecdotal data available to judge the efficacy of medical treatment in preventing growth in individual women or recurrence rates and the risks of repeat surgery. Today, clinical experience suggests avoiding or postponing surgery for cystic ovarian endometriosis of less than 3 cm unless there is severe pain. More data are needed in order to judge the treatment by transvaginal hydro-laparoscopy [45,46]. In women with severe pain and suspicion of deep endometriosis, a diagnostic laparoscopy and surgery should be carried out by a surgeon/group familiar with infertility and endometriosis surgery.

### 3.4. The Quality of Infertility Surgery Is Decreasing

**Figure 1:**
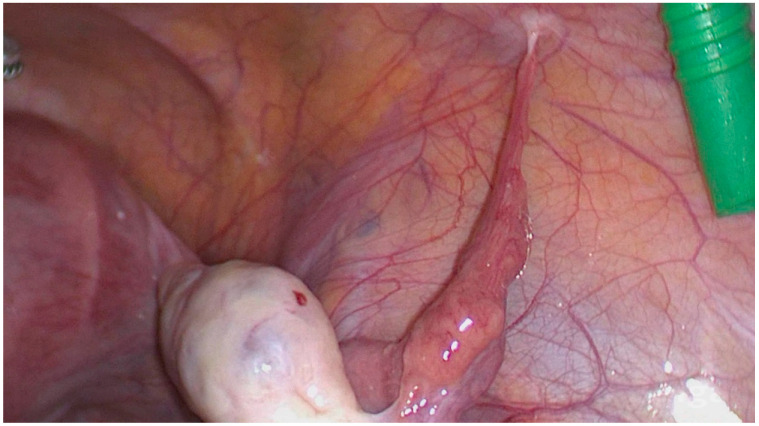
Adhesion between the ampulla and abdominal wall causing infertility would be missed without a laparoscopy. Surgery is uneventful and restores fertility.

Balancing infertility surgery and IVF is difficult because of the many variables. The choice should consider the results and risk of complications; the time to pregnancy; the woman’s age and antecedents; and the ultimate cumulative pregnancy rates, including subsequent pregnancies. Comprehensive data that consider all factors are unavailable, since most factors are not well-known and their values cannot be compared. Arguments that favour IVF before surgery are the absence of a risk of surgery and the perception that the time to pregnancy will be shorter. Arguments in favour of surgery are based on the clinically educated guess that without a systematic diagnostic laparoscopy during the infertility workup, some pathologies will remain undiagnosed and, thus, untreated. An extreme but rare example is two small filmy adhesions between the ampulla and abdominal wall that takes 10 s to cut without risk (Figure 1). In the absence of control groups, the fertility-enhancing effects of surgery are poorly established for most interventions, such as adhesiolysis and superficial, deep, or cystic ovarian endometriosis. Even for cystic ovarian endometriosis, it is challenging to balance the cumulative pregnancy rates, around 60% after surgery [47]; the ovarian damage; and the postoperative adhesions with the observation that surgery often does not improve the results of IVF. 

The major problem is that the quality of and indications for fertility surgery are difficult to evaluate. Historically, fertility surgery stimulated the development of microsurgery and laparoscopic surgery. Today, however, the surgical expertise of most infertility centres has decreased, and severe endometriosis surgery is increasingly performed by pelvic surgeons or in multidisciplinary teams with abdominal surgeons and urologists, who, unfortunately, are less experienced with fertility surgery. The clinical loss can be illustrated by thin-walled hydrosalpinges that can be treated with salpingostomy, salpingectomy, or IVF. Salpingostomy has been performed less often recently, since salpingoscopy to judge the tubal mucosa seems largely forgotten. Additionally, a salpingostomy requires either stitching, as was performed in the past by microsurgery, or a CO_2_ laser, which is rarely available today. Therefore, salpingectomies are increasingly performed since IVF results have improved after salpingectomy, although this is probably equally true following salpingostomy. Another example is the need for the preservation of ovarian tissue during surgery. Beyond the well-known decrease in follicular reserves after surgery [48], many have seen patients with significantly reduced ovarian volumes after surgery for rather small endometriomas.

This decrease in expertise in fertility surgery, together with the increasing age of the patients, is often presented as an argument in favour of the “IVF- first” approach. Unfortunately, in the absence of data documenting fertility enhancement and the quality of fertility surgery, only clinical experience remains. In women requiring surgery for pain, adhesion prevention by microsurgical principles retains its full importance [49,50]. To date, no reliable evidence has proven that deep endometriosis surgery in an asymptomatic patient improves fertility. The same is true for intraoperatively discovered asymptomatic (small) nodules. 

Experience also suggests avoiding repetitive IVF treatment in women with rectovaginal deep endometriosis nodules, since repetitive oocyte pickup through a nodule frequently results in very difficult surgery afterwards. 

### 3.5. The Dogma of the Complete Excision of Endometriosis

Complete excision of endometriosis has been a dogma of endometriosis surgery. The large bowel resections and excisions performed with a safety margin “to be complete” have been challenged by the non-progressive microscopical endometriosis nests in the bowel wall at a distance from nodules, in the lymph nodes and the peritoneum, explaining the similar recurrence rates after large bowel resections and conservative excisions despite most likely being microscopically incomplete. With the increased knowledge of the sympathetic nervous system, surgery has become less exhaustive when necessary to avoid functional sequelae. Although fibrosis surrounding endometriosis belongs to the body, resection of the fibrosis remains the overall standard. 

The clinical experience has, thus, resulted in more restraint when excising endometriosis with short bowel resections or wedge resections with a circular stapler, replacing extensive bowel resections, as more recent developments. However, it remains a personal judgment based on experience and personal skills, not on data, to balance the completeness of excision with functional sequelae and leaving some fibrosis. Similarly, it remains unclear whether the excision of large areas of the peritoneum for superficial endometriosis is beneficial or should be abandoned. 

### 3.6. The Shoe Shop Syndrome 

All shoe shops sell shoes, but only the shoes from their shop. Endometriosis management requires expertise in pain, infertility, medical therapy, and surgery. Unfortunately, these various aspects are organised in overlapping sub-specialities with specific meetings, societies, and journals. The result is that the diagnosis and treatment of endometriosis can vary according to the various disciplines. Especially for surgery, the exchange of knowledge is difficult, since surgery does not fit within the logic of evidence-based medicine with the RCT on top of the pyramid of evidence, and experience is considered a personal opinion of low value. Unfortunately, the variability of endometriosis and surgical skills, as well as the limited number of interventions, do not fit the RCT’s requirements [18]. 

Therefore, the collective experience of clinicians should be considered to develop experience-based management that integrates and complements evidence-based guidelines [33]. 

### 3.7. Emphasis on Evidence-Based Medicine without Recognising Experience

Diagnosis and treatment should be based on evidence. To avoid bias, evidence-based medicine emphasises the double-blind RCT and statistical significance. However, traditional statistics only calculate the probability that an observed effect, e.g., the efficacy of a drug, can be explained by chance and can, thus, only refute, but not confirm, a hypothesis. Estimating the probability that a hypothesis is true requires a different type of statistical inference or Bayesian statistics [24]. The latter is more similar to medical thinking, with the probability of all potential diagnoses and the risk of mistakes being refined progressively as more test results become available and as experience in surgery increases; each diagnosis and therapy is considered an experiment with an outcome. The clinician will “update” progressively by analysing and accepting what went well and where there was a challenge, in order to find a better way of achieving the result. For clinicians, it is important to grasp the relationship between traditional and Bayesian statistics. A *p*-value of 0.05 indicates a 5% probability that the effect can be explained by chance, but it is not an argument that the hypothesis is true. It does, however, change the probability that the hypothesis is correct from 50% to approximately 70% [51]. For the same reason, data that do meet statistical significance can be important, as illustrated by the fact that *p* = 0.05 and *p* = 0.06 are not very different.

EBM data on endometriosis are limited because of the absence of a helpful animal model permitting experimentation, as well as because of endometriosis lesions’ biochemical, macroscopical, and surgical variability. Most data on the efficacy of medical therapy can be questioned, since blinding is impossible when patients recognise the active treatment, e.g., when affecting menstruation. Moreover, the biochemical variability of the lesions invalidates traditional statistical analysis, since the essential assumption of a homogeneous population is not met. Continuing therapy despite incomplete pain relief comes close to the definition of madness, i.e., “repeating the same thing and expecting a different result”. Meaningful RCTs of deep endometriosis surgery are nearly impossible since the number of surgeries is limited, and the inherent variability of the disease would require large multivariate trials. Therefore, today, the clinical experience shared by many clinicians, which is sharpened by literature and congress discussions, is the best we have. This, however, needs to be developed in a more formal way [33].

This is another argument that the collective experience of clinicians should be considered in the experience-based management of endometriosis [33]. 

### 3.8. The Absence of Quality Control in Surgery

Medication must prove its efficacy and an absence of side effects to obtain a licence for marketing. This process is strictly organised into phase I, II, and III trials, as well as post-marketing surveillance. Quality control in surgery is indirect and varies between countries. Some countries limit the number of gynaecologists that can perform surgery (France, Germany); hospitals generally limit the number (Italy, UK) and the age of gynaecologists (e.g., Belgium) who can perform surgery; and limitations to privileges (USA) can restrict the type of intervention that each individual can perform. Although complication rates are registered, these reflect “the good, the bad and the ugly”, with quality control for an individual surgeon being limited to the poorly defined judgement of their peers. Periodic physical checks, although they are standard for many professions, such as pilots, and periodic checks on whether knowledge is up to date are not performed. This results in nearly unchecked freedom to use many different techniques, indications, and personal preferences, with few rules to stop a surgeon from performing surgery once board-certified. New techniques and materials are rarely properly evaluated before introduction, with laparoscopic surgery and the use of meshes as examples. The quality of surgery is not assessed since it is unclear which criteria should be used, but also because of the corporate opposition by the surgeons. A simple criterion, such as the excessive duration of surgery, is rarely considered, although it increases postoperative adhesions and the cost of surgery. Surgeons are opposed to, and governments do not implement, mandatory video registration [52,53,54], which could permit monitoring of the indications for surgery; individualise the billing of, e.g., severe endometriosis; and differentiate between mistakes, errors of judgment, and inevitable complications at a minimal cost. As an example, a ureter can be sectioned because of insufficient skills, either by mistake or intentionally, as part of the management of ureteral stenosis. In addition, mandatory video registration will improve quality through self-regulation, as it will encourage surgeons to be more prudent in order to avoid showing mistakes or a lack of expertise. 

These considerations call for the introduction of minimal quality control of the indications and techniques of surgery. Video registration could be a first step in introducing the principle of debriefing and learning from mistakes and near-misses without penalty, but with peer-reviewed quality control. 

### 3.9. Training and Education in Surgery Should Be Improved

Without quality control, it is unsurprising that education and surgical training are poorly defined. Today, the emphasis still lies on how to perform it and learning from examples. Recalling live surgery over the last 30 years, it seems important to change the tone from how to perform it to explaining the reasons behind each aspect of surgery. This significant shift would signify the acceptance of surgical dissection and nuances in tissue recognition and handling, bringing the focus to strategy and purpose. The fact that this is ultimately similar to understanding what is quality can be illustrated by examples. 

Surprisingly, suturing and knot security, which are fundamental in surgery, had been poorly investigated until recently [55]. Postoperative adhesions cause pain, infertility, and more difficult and complication-prone repeat surgeries [49]. However, adhesion prevention does not receive the attention it deserves. Most surgeons still use saline for irrigation, although it is known to be toxic for the peritoneum and to cause adhesions. However, blood causes adhesions [56], and fibrin can be challenging to remove at the end of the surgery. Irrigation is often avoided to facilitate dissection, even in minor surgery; gauzes, banned in microsurgery, are being reintroduced in laparoscopic surgery. 

This is another argument for quality control of the indications and techniques of surgery. Although machine learning and “safety” tools may help, intuition and experience need to be based on evidence, whether evidence-based or experience-based.

### 3.10. An Independent Expert Is a Rare Bird

When discussing the diagnosis of and therapy for endometriosis, we should realise that as humans, we live with the history of our past and with a potential bias in our judgment. It is rare to realise the difficulty and often blindness of cutting the branch on which we are sitting. Most people involved in medical therapy of endometriosis have or had ties with pharmaceutical companies, albeit as advisors, for clinical trials or as sponsors for congresses. Surgeons invariably also promote their practices in publications and live surgery. This is not a criticism, but we should be aware of potential biases, although most of us are looking for “the truth” in order to improve the care of our patients. 

## 4. Discussion

To the best of our knowledge, this manuscript is the first description of what should not be done or avoided in the management of endometriosis. To illustrate the importance of experience, we described our perception as surgery-oriented clinicians of the ten major problems in diagnosing and treating endometriosis based on the cumulative experience of the authors gained from over 50,000 women with endometriosis over more than 20 years in many different countries. These experience-based comments focused on what we perceived as the ten most important issues that should be avoided, taking into account rare events and mistakes. It should be clear that what should not be done cannot be investigated in a trial and that, therefore, these collective experience-based comments can only be complementary to EBM or surgical guidelines. However, today, these comments should be viewed as a Bayesian “Prior” for the calculation of the probability of truth when new data become available. 

These experience-based problems in the management of endometriosis illustrate the differences between research and clinical medicine. Research limits variability by inclusion and exclusion criteria in order to understand mechanisms. Clinical medicine is about the entire population, including multimorbidity and rare events. Another difference is that most medical decisions are multifactorial, whereas exploring many factors and their interaction is difficult in RCTs, albeit for randomisation issues [57]. Research and medicine are like the vertical and transversal columns of a table: the former describes the symptoms, e.g., pain, clinical investigation, imaging, and biochemical findings for each disease. The latter estimates the probabilities of many potential diagnoses for a woman with pain, as well as the consequences of missing a rare but important diagnosis, such as cancer. This is more similar to artificial intelligence [58]. This also illustrates the difficulty of implementing EBM and EBM-based guidelines [29,31,59]. They begin from research data and ignore experience, since it is considered to be of low value. However, to translate evidence into clinical guidelines, data need to be interpreted as grades of evidence by a group of specialists with different backgrounds. However, the use of experience to interpret data cannot be avoided. However, we suggest that data interpretation should only be performed by those with experience. Although the interpretation might vary between pain and infertility specialists and surgeons, surgery should be judged by surgeons. Experience should be documented and used in a manner complementary to EBM [32,60,61,62]. 

The major experience-based problems which we identified can be summarised as follows. Recognising the G-E and biochemical heterogeneity of endometriosis lesions is important to understand the variable response to medical therapy and the need for follow-up during treatment. The lower detection limit and the PPV of imaging outside referral centres should be recognised. Many problems result from sub-specialisation, misunderstanding EBM, and the absence of quality control in surgery. The common wisdom that “what we don’t know, we fear” explains the different perceptions of many aspects, including surgery complications. That frequentist or traditional statistics can only refute, but not confirm, a hypothesis is a mistake often made in medicine. Poor knowledge of Bayesian statistics prevents experience from being considered as a means of learning from mistakes and updating one’s knowledge, especially in surgery. Surgeons are also to be blamed for not organising quality control for surgery and its results. 

Only ten major problems in managing endometriosis were described, as perceived by the authors, who are deep endometriosis surgeons. Minor issues resulting from less expertise or the need for a more holistic approach, including sexual well-being [63,64], were not discussed. We also avoided items that can be addressed in RCTs or the integration and extrapolation of trial-based recommendations and classification systems. Therefore, these “cardinal sins” should be considered an invitation to document, discuss, or update experience in managing endometriosis by other groups or subspecialists. Following Bayesian validation of experience-based conclusions, their integration into EBM and surgical guidelines can be considered. 

## 5. Conclusions

Our discussion of the ten cardinal sins of surgery for endometriosis presents an experience-based view to complement evidence-based medicine for endometriosis. As significant aspects, we discussed understanding the pathophysiology of endometriosis, the interpretation of diagnostic tests, the limitations of EBM, the sub-disciplines, the importance of incorporating experience-based medicine, and the need for quality control in surgery.

## Data Availability

Before and after the meeting, over 300 questions were rated on a 0 to 10 visual analogue scale. Details of the answers are available by simple request to the corresponding author.
